# Predictors of Weight Gain in Individuals Living with HIV on Antiretroviral Therapy: A Cross-Sectional Study

**DOI:** 10.3390/nursrep16050159

**Published:** 2026-05-08

**Authors:** Thaynara Helena Ribeiro e Silva Medeiros, José de Ribamar Medeiros Lima Junior, Conceição de Maria Pedrozo e Silva de Azevedo, Larissa Di Leo Nogueira Costa, Marisa Cristina Aranha Batista, Francyelle Costa Moraes, Rachel Melo Ribeiro, Almir José Guimarães Gouveia, Ana Hélia de Lima Sardinha, Fernanda Costa Rosa, Mayara Soares Cunha, Leonel Lucas Smith de Mesquita, Luciana Batalha Sena, Sara Fiterman Lima, Angela Falcai

**Affiliations:** 1 Centro de Ciências Biológicas da Saúde, Universidade Federal do Maranhão, São Luís 65080-805, Brazil; jrml.junior@ufma.br (J.d.R.M.L.J.); conceicaopedrozo@ufma.br (C.d.M.P.e.S.d.A.); larissa.nogueira@ufma.br (L.D.L.N.C.); marisa.aranha@ufma.br (M.C.A.B.); francyelle.moraes@discente.ufma.br (F.C.M.); rachel.melo@ufma.br (R.M.R.); almir.guimaraes@ufma.br (A.J.G.G.); anahsardinha@ufma.br (A.H.d.L.S.); mayara.sc@ufma.br (M.S.C.); leonel.smith@ufma.br (L.L.S.d.M.); luciana.batalha@ufma.br (L.B.S.); sara.fiterman@ufma.br (S.F.L.); 2 Postgraduate Department Postgraduate Program in Bionorte, Universidad Uniceuma, São Luís 65075-120, Brazil; nandacosttarosa@gmail.com (F.C.R.); angelafalcaisceuma@ceuma.br (A.F.)

**Keywords:** HIV, antiretroviral therapy, body mass index, weight gain, comorbidities

## Abstract

**Objective:** To identify the predictors of weight gain in people living with HIV (PLHIV) using antiretroviral therapy (ART). **Methods:** Observational, analytical, cross-sectional study with a quantitative approach, with 186 participants followed up at a specialized HIV service. Sociodemographic, behavioral, clinical, and anthropometric data were collected. Associations between overweight and independent variables were assessed using chi-square and logistic regression analyses, adopting a significance level of *p* < 0.05. **Results:** Most participants were male (62.5%), aged between 40 and 49 years (33.2%). Overweight was prevalent in 70% of the sample, predominantly affecting males, sedentary individuals, and those with diabetes mellitus. There was a statistically significant association between overweight and age (*p* = 0.047), as well as with a diagnosis of diabetes (*p* = 0.029). Despite the high rate of viral suppression (97.3%), there was a prevalence of metabolic alterations, such as dyslipidemia and sedentary lifestyle. **Conclusions:** Weight gain in PLHIV in ART is associated with metabolic factors and age. The strategic role of health professionals in monitoring BMI, health education, and preventing unfavorable clinical outcomes, such as diabetes and cardiovascular diseases, is highlighted.

## 1. Introduction

The introduction of combination antiretroviral therapy in the 1990s provided HIV-positive individuals with reduced mortality, morbidity, and a higher quality of life [1]. By the year 2024, it was estimated that in Brazil, 82% of people who knew their HIV diagnosis had access to antiretroviral treatment, and 95% of these lived with a suppressed viral load [2].

Viral suppression, increased life expectancy, and reduced morbidity among HIV carriers also altered the nutritional and body profile of these patients. Unlike in the past, reports of weight loss, malnutrition, and cachexia after starting treatment are rare today; on the other hand, cases of overweight, obesity, and lipodystrophy have progressively increased [3,4].

Unlike the general population, weight gain in PLHIV is a multifactorial phenomenon. The virus induces a persistent inflammatory state that alters lipid and glucose metabolism, while certain classes of antiretroviral therapy (ART) interfere with adipocyte differentiation. This synergy increases the risk of insulin resistance and diabetes mellitus, creating a cycle where the metabolic disorder and the accumulation of adipose tissue feed each other, increasing the cardiovascular risk of this group [1,4,5].

People living with HIV who gain weight excessively are at higher risk of developing diabetes mellitus, cardiovascular diseases, non-defining cancers, hypertension, dyslipidemias, liver disease, neurological diseases, sarcopenia, orthopedic diseases, and rheumatological diseases [1,4,5].

As in the uninfected population, the prevalence patterns of weight gain vary in different countries and are impacted by several factors. The literature suggests that weight gain in HIV carriers is deeply influenced by a complex interaction of genetic and metabolic factors, direct effects of antiretrovirals, as well as sociodemographic and behavioral aspects, including environmental factors such as lifestyle and cultural factors [5,6,7].

The different classes of antiretrovirals have different metabolic impacts. While Protease Inhibitors (PIs) are historically linked to lipodystrophy and dyslipidemia, Integrase Inhibitors (INSTIs) have been linked to steeper and faster weight gain. These drugs can induce fat redistribution and changes in insulin signaling, increasing the risk of diabetes mellitus and cardiovascular disease, which makes BMI monitoring a clinical priority. However, some predictors have been shown to be quite consistent in several studies around the world, such as: low CD4 cell count, increased HIV viral load, female gender, black ethnicity, and integrase inhibitor-based ART regimens [3,4,6,8].

Weight gain in people living with HIV is an issue of utmost importance, recognized by the guidelines established by the European AIDS Clinical Society (EACS) and the treatment of obesity in patients with HIV requires a comprehensive approach that integrates pharmacological therapies, surgical strategies, and lifestyle changes, aiming to reduce cardiovascular risks and control weight effectively and safely [9,10,11].

The body mass index (BMI), when analyzed in isolation, presents limitations in predicting the health status of individuals, not providing detailed information on body composition, such as the proportion of adipose tissue and muscle mass, which are relevant aspects for the prevention and treatment of diseases. BMI measurement continues to be widely used in health services in Brazil due to its ease, low cost, speed, and high reproducibility, making it valuable for effective nutritional screening in extensive health systems, such as in Brazil [8,9,12].

Considering the risks that are strongly associated with simple weight gain in PLHIV individuals on antiretroviral treatment, there is a growing need to reinforce and expand the use of tools that facilitate early detection, weight control over time, surveillance of nutritional status, as well as to know the factors that can lead to weight gain in these individuals in a specific way [13]. Although global studies point to consolidated trends in weight gain during ART, the magnitude of this impact on populations in the Brazilian Midwest and Northeast regions still needs to be further detailed, given the influence of socioeconomic determinants and regional eating habits [14].

This study addresses an important gap by focusing on people living with HIV (PLHIV) from the Brazilian Northeast, a region that is underrepresented in the literature and characterized by distinct socioeconomic and lifestyle factors. Unlike previous studies that describe general patterns of weight gain during antiretroviral therapy (ART), this research examines the combined effect of aging and metabolic comorbidities as predictors of overweight in this population.

These findings contribute to a better understanding of context-specific risk factors and have practical implications for nursing care, particularly in the development of targeted interventions, continuous anthropometric monitoring, and strategies aimed at preventing metabolic complications in PLHIV.

The objective of this study was to characterize weight gain during antiretroviral therapy and to verify its associations with clinical, sociodemographic and lifestyle factors that impact this outcome. Thus, identifying the specific predictors of overweight in this cohort not only strengthens the national scientific literature, but also provides practical support for health care management, allowing the recognition of groups vulnerable to obesity and chronic non-communicable diseases associated with long-term HIV treatment.

## 2. Methods

This is an observational, analytical, cross-sectional study with a quantitative approach, reported in accordance with the Strengthening the Reporting of Observational Studies in Epidemiology (STROBE) guidelines. The study was conducted at a State Reference Hospital for Infectious and Parasitic Diseases in São Luís, Maranhão, Brazil, between May 2024 and February 2025.

This outpatient reference center provides specialized care to approximately 5239 people living with HIV (PLHIV). The study population consisted of 186 adult patients (aged 18 to 59 years), of both sexes, who were receiving follow-up care at the service. Participants were selected through consecutive non-probabilistic sampling.

To enhance representativeness and minimize selection bias, recruitment was carried out at three points within the service: the clinical screening area while patients awaited multidisciplinary consultations, the specialized pharmacy during medication dispensing, and the laboratory during routine sample collection.

The inclusion criteria were a confirmed diagnosis of HIV infection and continuous use of antiretroviral therapy for at least one year. Participants were also required to be clinically stable, with no hospitalizations in the previous year. Exclusion criteria included pregnancy and clinical conditions that could independently affect weight and metabolic parameters, such as tuberculosis, leishmaniasis, cancer, chronic kidney disease, liver disease, pulmonary disease, and neurological disorders.

Although a convenience sampling strategy was adopted, the adequacy of the sample size was evaluated in relation to the planned statistical analyses. For the multivariate logistic regression model, the sample met the widely accepted criterion of a minimum of 10 outcome events per independent variable (events per variable—EPV). Considering the prevalence of overweight observed in the study population, the number of outcome events was sufficient to support the inclusion of independent variables, ensuring the stability and reliability of the estimated associations.

Data collection was performed by a trained multidisciplinary team (two nurses, one nutritionist, and one physician) through individual interviews using a semi-structured questionnaire. Anthropometric measurements (weight and height) were obtained using a calibrated mechanical scale (Sanny^®^ (American Medical do Brasil Ltda, São Bernardo do Campo, Brazil), 200 kg capacity, 100 g precision) and a stadiometer, following standardized procedures. Body mass index was calculated as weight (kg) divided by height squared (m^2^) and classified according to World Health Organization criteria. For analytical purposes, it was categorized into “not overweight” and “overweight” (body mass index ≥ 25 kg/m^2^).

Clinical and laboratory data, including lipid profile, fasting blood glucose, and viral load, were obtained from medical records, considering tests performed within three months prior to data collection. Dyslipidemia was defined according to the Brazilian Guidelines on Dyslipidemia.

The variables analyzed included sociodemographic characteristics (sex, age group, education level, employment status, and sexual orientation), behavioral factors (physical activity, smoking, and alcohol consumption), and clinical variables (systemic arterial hypertension, diabetes mellitus, metabolic syndrome, time since HIV diagnosis, duration of antiretroviral therapy use, and viral load).

Categorical variables were described using absolute and relative frequencies. Missing data were described and handled using complete-case analysis. Associations between overweight and independent variables were assessed using Pearson’s chi-square test or Fisher’s exact test, as appropriate. Multivariate logistic regression analysis was performed to identify independent predictors of overweight, using a backward elimination method. Adjusted odds ratios and 95% confidence intervals were estimated, adopting a significance level of 5% (*p* < 0.05). All analyses were performed using SPSS version 22.0.

Potential sources of bias include the use of convenience sampling and the cross-sectional design, which may limit causal inference and introduce selection bias. However, recruitment at multiple points within the service was used to improve sample representativeness and mitigate this limitation.

This study was approved by the Research Ethics Committee of the Federal University of Maranhão (CAAE: 76097623.3.0000.5087; Opinion No. 6.752.52) and conducted in accordance with National Health Council Resolution No. 466/2012. All participants provided written informed consent.

## 3. Results

A total of 186 individuals participated in the study; the majority were male (62.5%, n = 117), aged between 40 and 49 years (33.2%, n = 61), and self-identified as brown (50.0%, n = 92); as well, 50% had a marital status of married (n = 92), and 43.7% of the sample completed high school (n = 87). As described in Table 1. Regarding behavioral variables and lifestyle habits, according to Table 2, 68.5% of the individuals living with HIV were classified as sedentary at the time of the survey (n = 126), 75.5% were nonsmokers (n = 139) and 84.2% did not consume alcohol on a regular basis (n = 157).

**Table 1:** Sociodemographic variables of people living with HIV in outpatient care in São Luís, Maranhão State, Brazil. 2024–2025.

Variables	n	%
**Gender**		
Women	69	37.5
Male	117	62.5
**Age group**		
20 to 29 years old	18	9.2
30 to 39 years old	48	25.5
40 to 49 years old	61	33.2
50 to ≥ 60 years old	59	32.1
**Race/color**		
White	42	22.3
Black	52	27.7
Brown	92	50.0
**Marital status**		
Married	92	50.0
Single	67	36.4
Stable union	4	2.2
Divorced	6	3.3
Widower	12	6.5
Missing Data	3	1.6
**Education**		
No formal education	4	2.2
Elementary School	68	37.0
High School	87	47.3
Higher Education	24	13.0
Missing Data	3	0.5

Source: Authors.

**Table 2:** Behavioral variables of people living with HIV in outpatient care in São Luís, Maranhão, Brazil 2024–2025.

Variables	n	%
Physical Activity *		
No	126	68.5
Yes	55	29.9
Missing Data	5	1.6
**Smoking**		
No	139	75.5
Yes	47	24.5
**Alcoholism**		
No	157	84.2
Yes	29	15.8

Source: Authors. * Note: Physical activity refers to regular practice of exercise. Regarding the clinical data of the individuals in the study, only 35.9% of the sample had a diagnosis of Systemic Arterial Hypertension (n = 66), 16.8% reported the onset of cardiovascular disease after the use of ART (n = 31), 45.7% of the interviewees had a diagnosis of diabetes mellitus (n = 54), 78.3% of the sample was composed of those who had been diagnosed with the disease between 1 and 10 years (n = 144), Viral load was undetectable in 97.3% of the sample (n = 181), in relation to the lipid profile, 69% had desirable cholesterol (n = 127), 64.7% had altered triglycerides (n = 120), and 62% had altered HDL (n = 115), as observed in Table 3.

**Table 3:** Clinical characteristics of people living with HIV receiving outpatient care in São Luís, Maranhão, 2024–2025.

Variables	n	%
**Systemic Arterial Hypertension**		
No	117	63.6
Yes	66	35.9
Missing Data	3	0.5
Cardiovascular disease		
No	150	81.5
Yes	31	16.8
Missing Data	5	1.6
DM *		
No	99	53.3
Yes	86	46.2
Missing Data	1	0.5
**Diagnosis time**		
Up to 10 years	144	78.3
More than 10 years	39	21.2
Missing Data	3	0.5
**Viral load**		
Undetectable	181	97.3
Detectable	5	2.7
**TC**		
Desirable	128	69.0
Changed	58	31.0
Missing Data	-	-
**TGC**		
Desirable	66	35.3
Changed	120	64.7
Missing Data	-	-
**HDL (High-Density Lipoprotein)**		
Desirable	64	34.2
Changed	115	62.0
Missing Data	7	3.8

Source: Authors. * Abbreviations: DM: Diabetes Mellitus; TC: Total Cholesterol; TGC: Triglycerides; HDL: High-Density Lipoprotein.

Among individuals classified as overweight, there was a predominance of males (70.4%, n = 81). The highest proportion of overweight was observed among participants aged 40–49 years (90.0% within this age group). In addition, most individuals self-identified as brown (65%, n = 60), were married (69%, n = 64), and had completed high school education (67.8%, n = 59). A high proportion of participants (71.4%) reported not engaging in regular physical activity. Associating overweight with sociodemographic, behavioral, and clinical variables (Table 4), a statistically significant association was found only for patients diagnosed with diabetes (*p *= 0.029) and for increasing age (*p *= 0.047).

Multivariate logistic regression, adjusted for sex and duration of ART use, confirmed that age and diabetes mellitus remain independent predictors of overweight. Patients with diabetes were over twice as likely to have a BMI ≥ 25 kg/m^2^ (OR: 2.21; 95% CI: 1.12–4.35; *p *= 0.029). Furthermore, individuals aged 40 to 49 years exhibited the highest risk profile for weight gain compared to younger cohorts (OR: 3.15; 95% CI: 1.05–9.48; *p *= 0.047), when compared to younger groups. It is noteworthy that, although a sedentary lifestyle had a high absolute frequency (71.4%), its strength of association did not reach statistical significance in the final adjusted model (*p* > 0.05), as mentioned in Table 5.

## 4. Discussion

In this study, a significant prevalence of overweight (overweight + obesity) was observed among the participants, as 70% of the individuals investigated had a BMI ≥ 25 kg/m^2^. This data corroborates the growing trend found in other studies, in which weight gain becomes significant in people living with HIV after starting ART [15,16,17,18].

Among overweight individuals, there was a predominance of men (70.4%), aged between 40 and 49 years (90%), self-declared brown (65%), married (69%) and with schooling up to high school (67.8%). In addition, most reported being sedentary (71.4%) and not consuming alcohol regularly (71.2%). According to the investigation by Augusto et al. [19], women under 40 years of age, of black ethnicity, using Dolutegravir and Tenofovir, showed more significant weight gain than men in the same circumstances.

Despite this, the statistical analysis showed a significant association only between overweight and the presence of diabetes mellitus (*p* = 0.029), as well as with increasing age (*p* = 0.047). The link between advancing age and weight gain in PLHIV is well-established, potentially driven by physiological shifts such as reduced basal metabolic rate and body fat redistribution. While sedentary behavior (71.4%) and time since diagnosis did not achieve statistical significance in the final model, their high clinical prevalence remains a critical factor. These elements contribute to a lifestyle-determined metabolic vulnerability that, when combined with aging and diabetes, complicates the clinical management of this population. These factors act as determinants of lifestyle, which, together with the predictors of age and diabetes, make up the metabolic vulnerability profile of the sample [20,21].

In a study conducted by Taramasso et al. [22], it was found that there is a worrying increase in the prevalence of comorbidities as the life expectancy of these individuals equals the life expectancy of the uninfected population. Another point found was that the accumulation of visceral adipose tissue, observed more frequently from the fourth decade of life, is associated with increased cardiovascular risk and insulin resistance in PLHIV [23].

Weight gain was also significantly associated with the presence of diabetes mellitus, which points to a worrisome metabolic profile in this population. Studies show that the risk of developing diabetes in PLHIV is increased, especially among those who are overweight or obese, have long-term use of ART, and have low physical activity [24,25]. In addition, certain classes of antiretrovirals, such as integrase inhibitors (INSTIs), have been strongly associated with glycemic changes and body fat accumulation, even in patients with an undetectable viral load [24,25,26,27].

An epidemiological study conducted on infected people in Europe and the US looked at the relationship between integrase inhibitor-induced weight gain and the development of diabetes in more than 20,000 people with HIV, followed for a median of just under five years. The study found that 785 participants (4%) were diagnosed with diabetes and that those taking an integrase inhibitor had a 48% higher risk of developing the disease, compared to other classes of antiretroviral drugs [28].

Although a sedentary lifestyle was highly prevalent among overweight participants (71.4%), there was no statistically significant association. However, the literature highlights that regular physical activity is a protective factor against weight gain, lipid alterations, and insulin resistance in PLHIV [20]. This reinforces the need for multidisciplinary strategies that encourage self-care and healthy lifestyle habits, especially among individuals at higher metabolic risk [27,29].

In the present study, 97.3% of the participants had an undetectable viral load, indicating virological efficacy of ART. However, this infection control does not prevent the occurrence of metabolic adverse effects, requiring continuous and integrated clinical monitoring [21]. In this context, the importance of the work of health professionals is highlighted, as they play a strategic role in the regular anthropometric follow-up of patients on ART, especially in the monitoring of BMI. Periodic measurement of weight and height, associated with clinical evaluation and individualized counseling, allows the early identification of excess weight and associated cardiometabolic risk factors [18,23,29].

In addition, the health team is a central piece in health education, guiding patients on healthy eating, physical activity, treatment adherence and prevention of comorbidities. The person-centered care strategy, widely adopted among health professionals, favors the construction of bonds, qualified listening, and personalized interventions, and is essential for strengthening patient autonomy and preventing unfavorable clinical outcomes such as diabetes, hypertension, and cardiovascular disease [18,23,29].

The findings of this study have direct implications for the clinical management of PLHIV, especially in the context of nutritional aspects. The identification of age (*p *= 0.047) and diabetes mellitus (*p *= 0.029) as factors associated with overweight signals the need for a paradigm shift: care must transcend viral load surveillance to focus on continuous metabolic health. For nurses, this implies the implementation of rigorous anthropometric monitoring protocols at each consultation, using not only BMI, but also abdominal circumference measurement as early indicators of cardiovascular risk [29].

This study has some limitations that should be considered. The cross-sectional design does not allow for causal inferences, and the use of convenience sampling may limit the generalizability of the findings. In addition, potential selection bias related to convenience sampling should be considered. Furthermore, the absence of detailed data on antiretroviral regimens and dietary patterns may have restricted a more in-depth analysis of factors associated with weight gain.

## 5. Conclusions

The results of this study indicate that, although ART is highly effective in virological control, the prevalence of overweight and the risk of diabetes mellitus in PLHIV represent new and complex clinical challenges. The association of weight gain with age and metabolic profile reinforces the need for health professionals to adopt a vigilant and preventive posture. It is concluded that comprehensive care for this population should go beyond viral suppression, integrating constant anthropometric monitoring and multidisciplinary educational interventions aimed at lifestyle, aiming to reduce morbidity and mortality due to cardiometabolic causes and promote healthy aging. From an epidemiological point of view, the findings reinforce the transition in the morbidity and mortality profile of people living with HIV in the contemporary scenario.

The correlation observed between the accumulation of body fat, advancing age and the diagnosis of diabetes suggests that biological aging, added to the metabolic alterations typical of the chronicity of the infection, acts as a potent catalyst for weight gain. This indicates that immunovirological stability, although essential, is not sufficient to guarantee full health, requiring strategies that consider the complex interaction between the virus, the therapy used, and the biological determinants of the host.

It is concluded that age and diabetes mellitus are predictors of overweight in PLHIV. These findings highlight the need for nursing care protocols that integrate periodic anthropometric assessment with metabolic disease prevention programs, ensuring that the success of antiretroviral therapy is not compromised by comorbidities associated with weight gain. Future investigations, preferably of a longitudinal nature, are essential to evaluate the influence of new therapeutic classes on body composition and to test intervention models that can mitigate the progression of excessive weight, ensuring that the increase in life expectancy is supported by favorable metabolic indicators and a lower burden of associated comorbidities.

## Figures and Tables

**Table 4:** Sociodemographic, behavioral, and clinical variables according to the BMI of people living with HIV in outpatient care in São Luís, Maranhão.

	BMI		
Variables	No Overweight	Overweight	*p*-Value
	n (%)	n (%)	
**Sex**			
Women	22 (30.4)	49 (69.6)	1.000 a
Male	34 (29.6)	81 (70.4)	
**Age group**			
20 to 29 years old	2 (11.8)	15 (88.2)	**0.047 b**
30 to 39 years old	17 (36.2)	30 (63.8)	
40 to 49 years old	6 (10.0)	54 (90.0)	
50 to ≥60 years old	10 (20.0)	42 (80.0)	
**Race/color**			
White	9 (22.0)	32 (78.0)	0.297 a
Pardo (Mixed-race) *	14 (27.5)	37 (72.5)	
Black	32 (34.8)	60 (65.2)	
**Marital status**			
Married	28 (30.4)	64 (69.6)	0.995 b
Single	21 (31.3)	46 (68.7)	
Stable union	1 (25.0)	3 (75.0)	
Divorced	2 (33.3)	4 (66.7)	
Widower	3 (25.0)	9 (75.0)	
**Education**			
No formal education	-	4 (100.0)	0.273 b
Elementary School	18 (26.1)	51 (75.0)	
High School	28 (32.2)	59 (67.8)	
Higher Education	10 (41.7)	14 (58.3)	
**Physical Activity**			
No	37 (28.6)	91 (71.4)	0.700 a
Yes	19 (32.7)	39 (67.3)	
**Smoking**			
No	40 (28.8)	99 (71.2)	0.694 a
Yes	15 (33.3)	40 (66.7)	
**Alcoholism**			
No	48 (29.7)	109 (70.3)	1.000 a
Yes	9 (31.0)	20 (69.0)	
**Systemic Arterial Hypertension**			
No	38 (30.8)	81 (69.2)	0.910 a
Yes	19 (28.8)	48 (71.2)	
**Cardiovascular Diseases**			
No	49 (31.3)	105 (68.7)	0.693 a
Yes	8 (25.8)	25 (74.2)	
**DM (Diabetes Mellitus)**			
No	39 (37.4)	62 (62.6)	**0.029 a**
Yes	19 (21.4)	66 (78.6)	
**Diagnosis time**			
Up to 10 years	43 (29.2)	104 (70.8)	0.786 b
More than 10 years	13 (33.3)	26 (66.7)	
**Viral load**			
Undetectable	51 (29.6)	119 (70.4)	0.773 b
Detectable	5 (33.3)	11 (66.7)	
**Total Cholesterol**			
Desirable	40 (31.5)	88 (68.5)	0.592 a
Changed	15 (26.3)	43 (73.7)	
**Triglycerides**			
Desirable	18 (27.7)	47 (72.3)	0.754 a
Changed	39 (31.1)	82 (68.9)	
**HDL**			
Desirable	19 (30.2)	44 (69.8)	1.000 a
Changed	34 (29.8)	89 (70.2)	

Source: Authors. Pearson’s chi-square. Abbreviations: BMI: Body Mass Index; DM: Diabetes Mellitus; TC: Total Cholesterol; TGC: Triglycerides; HDL: High-Density Lipoprotein. * Ethnicity/Skin color was self-reported according to the categories used by the Brazilian Institute of Geography and Statistics (IBGE). ‘Pardo’ refers to multiracial/mixed-race individuals. a: Pearson’s chi-square test; b: Fisher’s exact test.

**Table 5:** Logistic regression analysis of factors associated with overweight in people living with HIV. São Luís, Maranhão, Brazil, 2024–2025.

Variables	Crude OR (IC 95%)	*p*-Value	Adjusted OR * (95% CI)	*p*-Value
**Diabetes Mellitus**				
No	1.00		1.00	
Yes	2.18 (1.15–4.12)	0.029	**2.21 (1.12–4.35)**	**0.029**
**Age Group**				
20 to 29 years old	1.00		1.00	
30 to 39 years old	1.22 (0.45–3.32)	0.680	1.18 (0.40–3.45)	0.720
40 to 49 years old	3.10 (1.12–8.55)	0.047	**3.15 (1.05–9.48)**	**0.047**
50 to ≥60 years old	2.15 (0.85–5.42)	0.102	2.05 (0.78–5.35)	0.145
**Sex**				
Women	1.00		1.00	
Male	1.04 (0.54–2.01)	1.000	1.02 (0.50–2.05)	0.945

Source: Authors. * Analysis adjusted for sex and time of ART use. OR: Odds Ratio; 95% CI: 95% Confidence Interval. Hosmer-Lemeshow test: *p *= 0.842. Abbreviations: OR: Odds Ratio; CI: Confidence Interval. Adjusted for sex and duration of antiretroviral therapy.

## Data Availability

The data presented in this study are available on request from the corresponding author. The data are not publicly available due to privacy and ethical restrictions, as the dataset contains sensitive clinical information regarding people living with HIV (PLHIV), in accordance with the Institutional Review Board (IRB) approval and the Brazilian National Health Council.

## Public Involvement Statement

Patients and the public were not involved in the design, conduct, reporting, or dissemination plans of this research.

## Guidelines and Standards Statement

This study was reported in accordance with the Strengthening the Reporting of Observational Studies in Epidemiology (STROBE) guidelines, and the corresponding checklist was completed to ensure transparency and completeness of reporting.
